# Expression of *Pinellia pedatisecta* Lectin Gene in Transgenic Wheat Enhances Resistance to Wheat Aphids

**DOI:** 10.3390/molecules23040748

**Published:** 2018-03-24

**Authors:** Xiaoliang Duan, Qiling Hou, Guoyu Liu, Xiaomeng Pang, Zhenli Niu, Xiao Wang, Yufeng Zhang, Baoyun Li, Rongqi Liang

**Affiliations:** Key Laboratory of Crop Heterosis and Utilization (MOE)/Beijing Key Laboratory of Crop Genetic Improvement, College of Agronomy and Biotechnology, China Agricultural University, Beijing 100193, China; dxl@chinagrain.org (X.D.); wheatqilinghou@163.com (Q.H.); guoyuliu16@163.com (G.L.); pxm382078070@163.com (X.P.); niuzhenlicau@163.com (Z.N.); wangxiaocau@163.com (X.W.); zhangyufeng870810@163.com (Y.Z.); baoyunli@cau.edu.cn (B.L.)

**Keywords:** *Pinellia pedastisecta*, *ppa*, lectin, wheat aphid, aphid resistance

## Abstract

Wheat aphids are major pests during the seed filling stage of wheat. Plant lectins are toxic to sap-sucking pests such as wheat aphids. In this study, *Pinellia pedatisecta* agglutinin (*ppa*), a gene encoding mannose binding lectin, was cloned, and it shared 92.69% nucleotide similarity and 94% amino acid similarity with *Pinellia ternata* agglutinin (*pta*). The *ppa* gene, driven by the constitutive and phloem-specific ribulose bisphosphate carboxylase small subunit gene (*rbcs*) promoter in pBAC-rbcs-ppa expression vector, was transferred into the wheat cultivar Baofeng104 (BF104) by particle bombardment transformation. Fifty-four T_0_ transgenic plants were generated. The inheritance and expression of the *ppa* gene were confirmed by PCR and RT-PCR analysis respectively, and seven homozygous transgenic lines were obtained. An aphid bioassay on detached leaf segments revealed that seven *ppa* transgenic wheat lines had lower aphid growth rates and higher inhibition rates than BF104. Furthermore, two-year aphid bioassays in isolated fields showed that aphid numbers per tiller of transgenic lines were significantly decreased, compared with wild type BF104. Therefore, *ppa* could be a strong biotechnological candidate to produce aphid-resistant wheat.

## 1. Introduction

Common wheat is the second most important food crop in China, and Northern Chinese people eat many wheat flour foods, such as noodles, steamed bread, and dumplings. Wheat aphids are devastating pests affecting wheat production, causing severe yield losses and significantly reducing crop quality each year [1,2,3]. There are four main species of wheat aphids (*Metopolophium dirhodum*, *Schizaphis graminum*, *Rhopalosiphum padi*, and *Sitobion avenae*) that are widely distributed in wheat-production areas in China. Every year, wheat-production fields with more than 1500–2500 aphids per hundred plants was up to from 15 to 20 million hectares, and the yield dropped by more than 10% (http://www.natesc.moa.gov.cn). Until now, aphid control has depended mainly on spraying large amounts of insecticides, causing considerable environmental pollution and increasing costs of wheat production. In order to breed aphid-resistant wheat cultivars, considerable effort has been made in searching for aphid-resistant genes and germplasm resources, and developing aphid-resistant wheat varieties [4,5,6,7,8].

Lectins, the carbohydrate-binding proteins found in most plants, play a role in protecting plants against external pathogens, insects, and other organisms. At present, many plant lectins have been introduced to transgenic plants to enhance resistance towards many sap-sucking insects [4,5,6,7,8]. *Galanthus nivalis* agglutinin (GNA), the mannose-specific lectin from snowdrop, has been shown to exhibit anti-metabolic effects in transgenic plants against many economically important homopteran pests, causing reduced survival and delayed development [9,10,11]. A GNA expression level of 0.3–0.4% of the total soluble protein in transgenic potato plants provoked a significant reduction in both survival and fecundity of potato aphid (*Aulacothum solani*) in greenhouse bioassays [12]. In addition to GNA, several GNA-like lectins from other monocots and their application in transgenic aphid-resistant plants have also been reported, including: Chinese *Narcissus tazetta* lectin (NTL); *Pinellia ternata* agglutinin (PTA); and *Arisaema heterophyllum* agglutinin (AHA) [13,14,15,16,17,18,19]. For example, PTA confers enhanced resistance to peach potato aphid (*Myzus persicae* Sulzer) in transgenic tobacco [15,16]. Duan et al. [20] synthesized *GNA* (*sGNA*) and *NTL* (*sNTL*) genes according to the codons preferable principle of wheat genes, and found that the transgenic lines were highly resistant to aphids compared to the corresponding control plants in the lab and in a two-year field bioassay.

Two novel mannose-binding lectin genes from *Arisaema heterophyllum* and *Pinellia tenata* of the family *Araceae* were cloned and introduced to tobacco, potato and wheat; both lectin genes enhanced resistance to aphids [13,14,15,16,17,18,19]. Pedate Pinellia (*Pinellia pedatisecta* Schott), is a traditional Chinese medicine that has long been used to cure thanatophidia bite, nameless swelling and toxicum, and cancer [21]. However, no information is available for *P. pedatisecta* agglutinin (PPA) genes and their effects on aphids in transgenic wheat plants.

In this study, the *ppa* gene was cloned using *P. pedatisecta* seedling RNA by homology cloning strategy, and driven by the constitutive and phloem-specific ribulose bisphosphate carboxylase small subunit (rbcs) promoter in the expression vector. The *ppa* gene was transferred into wheat cultivar Baofeng104 (BF104) by particle bombardment. The objectives of this study were: (1) to obtain the novel potential lectin gene; (2) to evaluate resistance to aphids indoors as well as in field conditions; and (3) to develop aphid-resistant wheat germplasm.

## 2. Results

### 2.1. Cloning of the ppa Fragment and Construction of the Expression Vector

**Figure 1:**
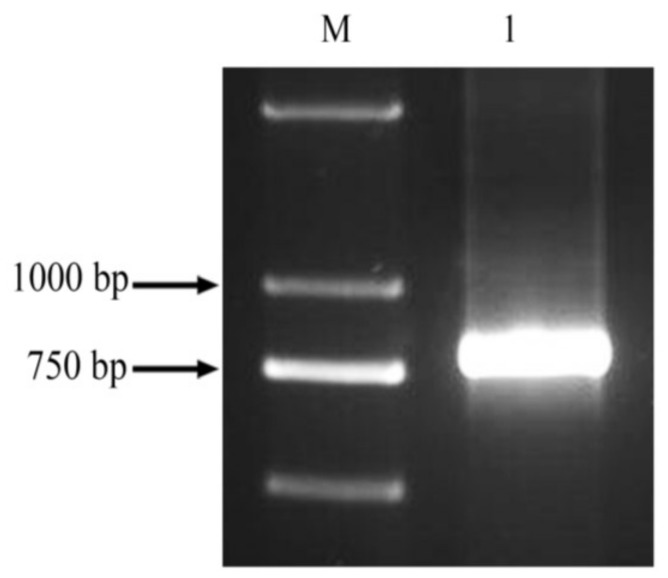
The products obtained by RT-PCR M: D2000; 1: *ppa* fragment.

**Figure 2:**
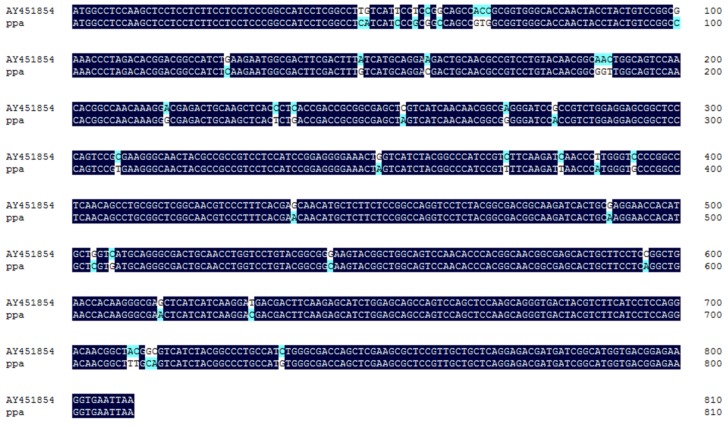
Nucleotide homology of *ppa* and *pta*.

As showed in Figure 1, the 0.8 kb fragments of the *ppa* gene (Figure 1) were amplified using RT-PCR with primers designed according to the lectin homolog in *Pinellia ternata* (*pta*). Nucleotide sequence alignment showed that the ORF of *ppa* shares 92.69% similarity with *pta* (GenBank No. AY451854); only 41 bases differed between the two sequences (Figure 2).

**Figure 3:**
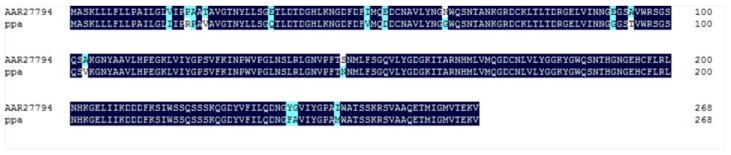
The predicted amino acid homology of PPA and PTA.

The *ppa* predicted amino acid sequence (PPA) shares about 94% similarity with the PTAs (GenBank Nos. ABX47148, AA205446, AAU29612, AAP20876, and AAR27794) from *P. ternata* (Figure 3), and 78.1% similarity with AHAs (GenBank Nos. AAP50524 and AAQ16181) from *Arisaema heterophyllum*. Those lectins share the same representative functional domains: ‘mannose-binding site’, ‘Bulb-type mannose-specific lectin domain’, and ‘dimerization interface site’.

**Figure 4:**
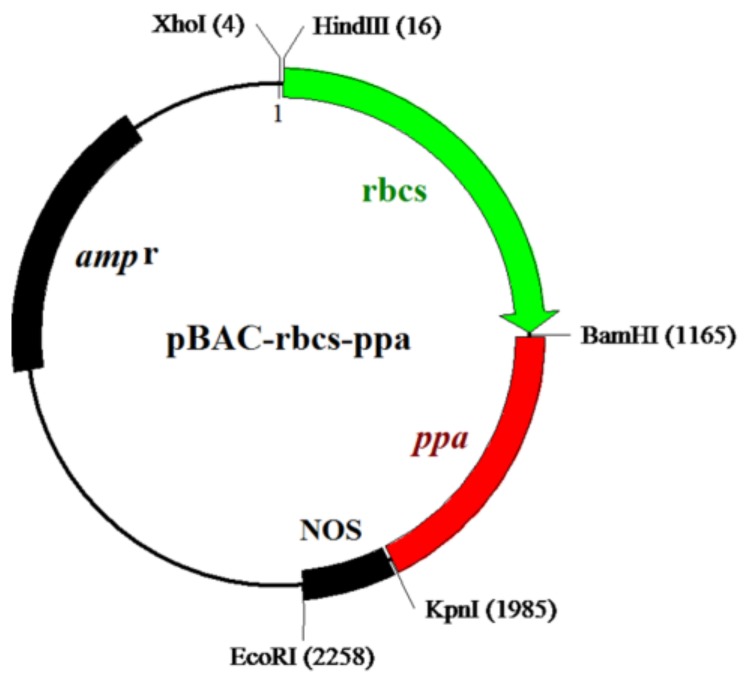
Schematic construction of the recombinant pBAC-rbcs-ppa vector.

The *ppa* fragment was cloned into the pBAC-rbcs-CbE E4 vector (Xu et al., 2014) by *Bam*H I and *Kpn* I digestion and ligation, generating the expression vector pBAC-rbcs-ppa (Figure 4).

### 2.2. Production of Transgenic Lines and Expression of the ppa Gene

**Figure 5:**
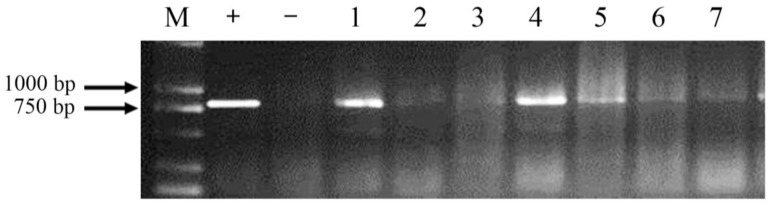
PCR detection of T_2_ transgenic lines M: D2000; +: pBAC-rbcs-ppa; −: BF104; 1–7: Transgenic lines BF1-4, BF1-16, BF2-7, BF6-6, BF6-12, BF18-1 and BF18-9.

Fifty-four regenerated (T_0_) plantlets were obtained; 34 of them were found to be positive transformants via PCR screening using the specific primers ppa-L2 and ppa-R2. In every generation, PCR-positive plants were selected and sown using the pedigree method to obtain homozygous transgenic lines. Seven T_2_ lines (BF1-4, BF1-16, BF2-7, BF6-6, BF6-12, BF18-1, and BF18-9, named after the field location of T_0_ plants in the greenhouse), derived from seven independent T_0_ plants, were positive for the *ppa* gene (Figure 5), but negative for the *bar* (biolaphos resistance gene).

**Figure 6:**
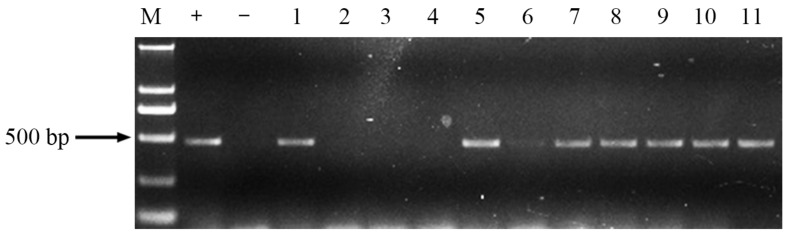
Expression of *ppa* of T_2_ transgenic lines by RT-PCR M: D2000; +: pBAC-rbcs-ppa; −: blank control; 1: *Pinellia pedatisecta* cDNA; 2, 3: grain cDNA of transgenic lines BF1-4 and BF1-16; 4: leaf cDNA of BF104; 5–11: leaves cDNA of transgenic lines BF1-4, BF1-16, BF2-7, BF6-6, BF6-12, BF18-1 and BF18-9.

Expression of *ppa* in leaves of transgenic wheat lines was checked using two-step RT-PCR with specific primers ppa-L3 and ppa-R3 designed using the *ppa* sequence (Figure 6). The results showed that the 460 bp bands were amplified, indicating that the *ppa* gene was expressed in leaves of seven T_2_ transgenic wheat lines. 

### 2.3. Aphid Bioassay on Detached Leaf Segments

**Figure 7:**
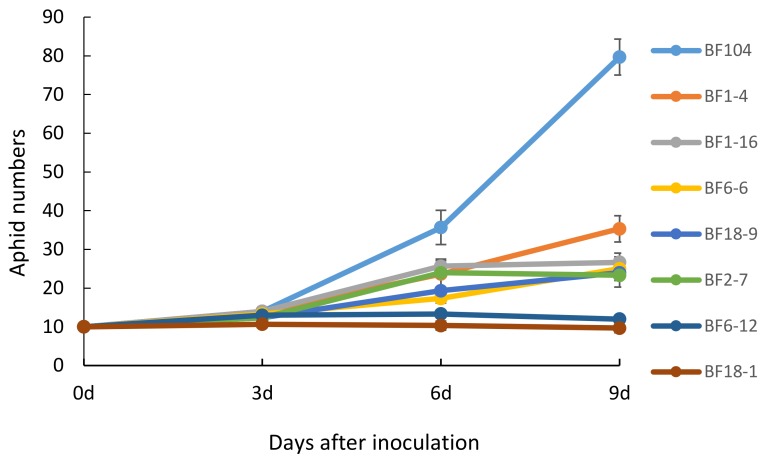
Numbers of aphids feeding on transgenic lines and on the control (BF104).

The seven transgenic lines were selected to evaluate aphid resistance using detached leaves (Figure 7). At the beginning of aphid inoculation, there was no difference in aphid number between transgenic lines and control (BF104); after 6 d, the aphid numbers on control leaves clearly increased while numbers in transgenic lines went up more slowly. After 9 d, the difference in aphid numbers was significant. Two lines, BF18-1 and BF6-12, exhibited the highest suppression on aphid fecundity. These results showed that *ppa* conferred aphid resistance to transgenic wheat lines.

**Figure 8:**
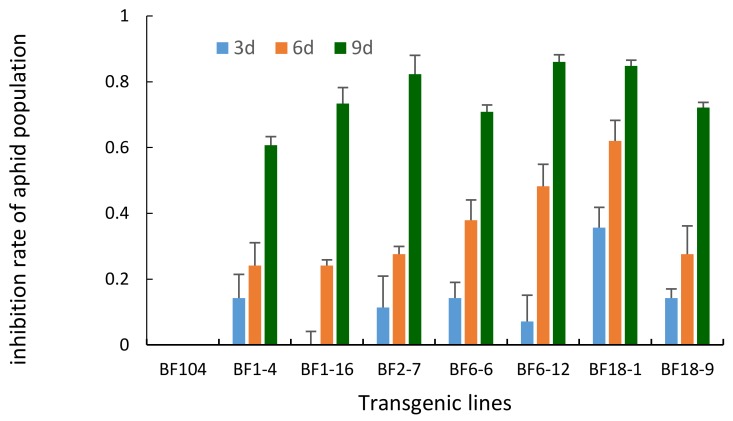
The inhibition rates of aphid population feeding on transgenic lines. The histogram bars represent the mean values and the error bars indicate standard deviations.

The inhibition rates of aphid population development from the aphid bioassay also showed that the transgenic lines were aphid-resistant (Figure 8). The rates of aphid population inhibition of transgenic lines were from 27.7 to 61.1% on day 6, and from 50.5 to 87.7% on day 9. Resistance to aphids differed among transgenic lines, probably due to different expression levels of *ppa*.

### 2.4. T_4_ and T_5_ Transgenic Wheat Lines in the Field Showed Lower Aphid Numbers

**Figure 9:**
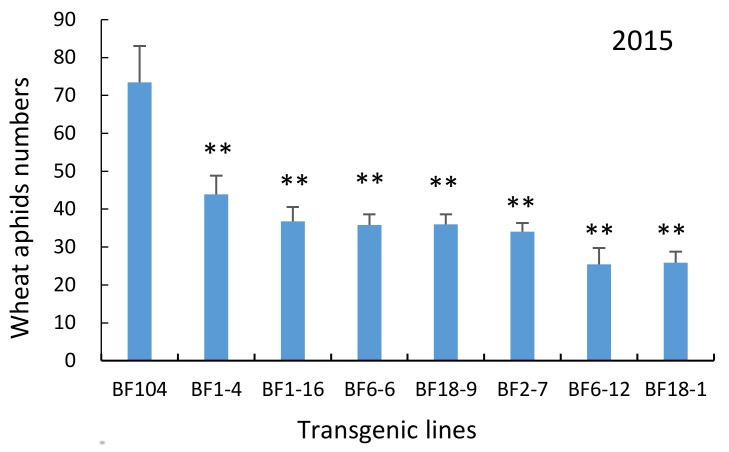
Wheat aphid numbers on transgenic lines under field conditions. The histogram bars represent the mean values and the error bars indicate standard deviations. The significance in the differences between transgenic plants and control plants were determined by the independent samples *t*-test at *p* < 0.05 or *p* < 0.01.

**Figure 10:**
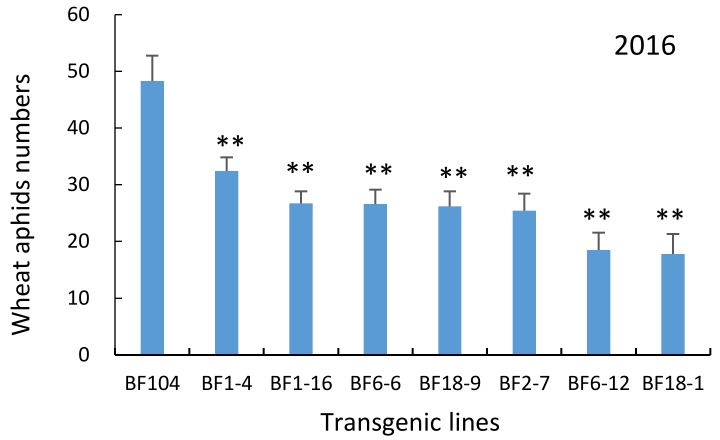
Wheat aphid numbers on transgenic lines under field conditions. The histogram bars represent the mean values and the error bars indicate standard deviations. The significance in the differences between transgenic plants and control plants were determined by the independent samples *t*-test at *p* < 0.01.

The numbers of natural populations of wheat aphids (four species) per tiller were counted on ten selected plants per block at 15 days after flowering (DAF). In 2015, aphid numbers per tiller on T_4_ transgenic wheat lines under field conditions were significantly reduced from 40.3 to 65.4% compared with the non-transgenic BF104 (Figure 9). In 2016, the aphid numbers on the field-grown T_5_ transgenic wheat line were significantly reduced from 32.9 to 62.1% compared with non-transgenic BF104 plants (Figure 10). The mean inhibition rate of seven lines in the two-year field assays was about 50%, showing that all transgenic lines had certain inhibitory effects on the growth and reproduction of wheat aphids, and the aphid-resistance levels of transgenic lines were different.

## 3. Discussion

Currently, many mannose-specific lectins have been shown to have a strong toxic effect on sap-sucking insects, and have been introduced to transgenic plants to enhance insect resistance [4,5,6,7,8,9,10,11,12,13,14,15,16,17,18,19]. When the GNA expression level was 0.3–0.4% of the total soluble protein, the potato aphid (*Aulacothum solani*) fed on transgenic potato plants exhibited significantly reduced survival and fecundity in greenhouse bioassays [12]. The inhibition rates of grain aphid (*Sitobion avenae*) were from 40 to 65% after feeding on GNA-expressing transgenic Bobwhite plants [22]. Liang et al. [23] obtained eight GNA-expressing aphid-resistant transgenic wheat lines with inhibition rates of about 47%. PTA conferred enhanced resistance to peach potato aphid (*Myzus persicae* Sulzer) in transgenic tobacco [15]. In our study, the *Pinellia pedatisecta* agglutinin (*ppa*) gene was cloned according to the lectin homolog in *P. ternata* agglutinin (*pta*), and its coding sequence shared 92.69% similarity with *pta*; further transformation of wheat showed that the inhibition rates of seven lines were from 50.5 to 87.7% after the 9 d bioassay on detached leaves and about 50% in two-year field assays, indicating that all transgenic lines had certain inhibitory effects on the growth and reproduction of wheat aphids. In our study, the rbcs promoter from tomato was used to drive *ppa*, therefore the *ppa* mRNA should be mainly found in leaf, instead of grain. Our results showed that there was no *ppa* detected in the grain 15 DAF (Figure 6: lanes 2 and 3), suggesting that no additional lectins affect edible mature grain for human beings. Therefore, *ppa* could be a strong biotechnological candidate, comparable to GNA and PTA, for use in efforts to produce aphid-resistant plants. 

## 4. Materials and Methods

### 4.1. Plant Materials and Aphids

Pedate Pinellia (*Pinellia pedatisecta* Schott), a species in the genus *Pinellia*, family *Araceae*, was provided by Professor Xingcui Zhang from Southwest University, Chongqing, China.

The winter wheat cultivar ‘Baofeng 104’ (BF104), a high-yield cultivar bred by the Chinese Academy of Agriculture (Beijing, China), was used as the receptor for transformation. Common wheat varieties ‘Jing411’ (J411), known to be susceptible to grain aphids, were bred by the Beijing Seed Corporation (Beijing, China). 

The monoclonal grain aphid (*Sitobion avenae*) population used for the indoor bioassay was raised in an artificial climate chamber with conditions of 21 ± 1 °C, 50–60% relative humidity, and with a 16 h-day/8 h-night cycle. The larvae were fed on 2-week-old J411 seedlings.

Seeds of the transgenic wheat lines and control (BF104) were surface sterilized in 1% sodium hypochlorite solution for 10 min, rinsed five times with distilled water, and grown in pots containing sterilized soil. The transgenic and control seedlings were grown in an environmental growth room under the same standard conditions mentioned above.

### 4.2. Gene Cloning and Sequencing of the ppa Gene Fragment

Total RNA was extracted from *P. pedatisecta* seedlings using TRIzol Reagent, and RNA concentration and purity were evaluated with a NanoDrop 2000 spectrophotometer (Thermo Scientific Branch, Beijing, China). The primers ppa-L1 (5′-GGATCCATGGCCTCCAAGCTCC-3′, the underlined letters are the *Bam*HI enzyme site) and ppa-R1 (5′-GGTACCTTAATTCACCTTCTCCG-3′, the underlined letters are the *Kpn* I enzyme site) were designed according to *P. ternata* lectin (GenBank AY451854). The *P. pedatisecta* cDNA was synthesized with M-MLV Reverse Transcriptase System (Promega Branch, Beijing, China) in accordance with the manufacturer’s instructions. The *ppa* gene fragments were amplified from *P. pedatisecta* cDNA by PCR with primers ppa-L1 and ppa-R1 with a 2 × Taq PCR MasterMix Kit (TaKaRa Branch, Beijing, China). The PCR cycling program was as follows: 94 °C for 5 min, followed by 35 cycles of 94 °C for 30 s, 60 °C for 1 min, and 72 °C for 1 min, and a final extension step at 72 °C for 10 min. PCR products were analyzed on 1.5% agarose gels. The purified fragments were cloned into the pGEM-T easy vector (Promega Branch, Beijing, China) and transformed into *E. coli DH5α* cells. The positive clones were sequenced commercially to verify the size and sequence of inserts. The sequence similarities between the *ppa* fragments and the reported *ptα* gene (GenBank AY451854) were determined by aligning the sequences.

### 4.3. Construction of Expression Vector pBAC-rbcs-ppa and Production of Transgenic Wheat

The *ppa* fragments were inserted into the pBAC-rbcs-CbE E4 vector [24] by *Bam*H I and *Kpn* I digestion and ligation, yielding the expression vector pBAC-rbcs-ppa. The *ppa* expression box contained an *rbcs* promoter, *ppa* gene fragment, and a NOS terminator.

pBAC-rbcs-ppa and pBAC35SIH3 (containing the *bar* gene, which encodes an herbicide resistance protein) [24] were mixed at the rate of 3:1 and cotransformed into wheat cultivar BF104 callus using the particle bombardment method. After selection by BASTA and differentiation, fifty-four regenerated plantlets were obtained, and these T_0_ plants and their offspring were further screened by PCR.

### 4.4. PCR and Screening of Homozygous Transgenic Lines

Each transgenic plant in every generation was screened by PCR and sown using the pedigree method (the seeds from one positive plant were sown into one row in next generation) until positive homozygous lines were obtained. The genomic DNA was extracted from leaf tissue using the CTAB method. For PCR analysis, the specific primers ppa-L2 (5′-ATGGCCTCCAAGCTCCTCCTC-3′) and ppa-R2 (5′-TTAATTCACCTTCTCCGTCACC-3′) were designed for the *ppa* gene. Additional primers were used for the *bar* gene screening. The DNA from non-transgenic plants and plasmids of pBAC-rbcs-ppa were used as negative and positive controls respectively. The PCR cycling program was as follows: 94 °C for 5 min, followed by 35 cycles of 94 °C for 30 s, 57 °C for 45 s, and 72 °C for 1 min, and a final extension at 72 °C for 10 min. PCR products were analyzed on 1.0% agarose gels.

### 4.5. Expression of ppa in Transgenic Wheat

RT-PCR was used to identify expression of the *ppa* gene in the transgenic wheat lines. Total RNA was extracted from flag leaves and grains on 15 DAF using the Trizol method, and then treated with DNase I (Invitrogen Branch, Beijing, China) prior to cDNA synthesis. The cDNA was made as described above. The specific primers ppa-L3 (5′-AGGGCGAACTCATCATCAAGGAC-3′) and ppa-R3 (5′-ATTCACCTTCTCCGTCACCATGCC-3′) were designed for the *ppa* gene, and the products were 460 bp. The *P. pedatisecta* cDNA and plasmids of pBAC-rbcs-ppa were used as positive controls, while the cDNA from non-transgenic BF104 plants as used as a negative control. The PCR cycling program was as follows: 94 °C for 5 min, followed by 35 cycles of 94 °C for 30 s, 56 °C for 30 s, and 72 °C for 45 s, and a final extension at 72 °C for 10 min. PCR products were analyzed on 1.0% agarose gels.

### 4.6. Aphid Bioassay on Detached Leaf Segments

Apterous adult grain aphids were placed in nylon cages on fresh J411 plants for 24 h to produce neonate nymphs for the aphid bioassay on 7 homozygous positive T_2_ transgenic wheat lines and on control BF104. The leaves of two-week-old seedlings in an environmental growth room were cut into 4 cm long pieces and inoculated in 13% agar medium containing 0.8% benzimidazole. Ten neonate nymphs were inoculated on the detached segments of each Petridish and inoculated at 24 °C with 3 independent biological replications. Aphid survival and fecundity were measured every three days (by parthenogenetic production of offspring) over a 9 d assay period under illumination incubator conditions (25 °C). 

The formula for calculating aphid resistance is as follows [22]: inhibition rate of aphid population development (%) = (aphid number in control plants−aphid number in transgenic plants)/aphid number in control plants × 100%.

### 4.7. Aphid Resistance Bioassay under Field Conditions

Aphids were counted on 15 DAF in an isolated experimental field at China Agricultural University, Beijing. Each transgenic wheat line and control (BF104) was planted in 3 rows with 2 m length and 0.25 m spacing in a random block design with three replicates. The numbers of mixed populations of wheat aphids per tiller were counted on ten plants per block selected by the Fuzzy Recognition method [25,26,27]. Any significance in the differences between transgenic plants and control plants were determined by the independent samples *t*-test at *p* < 0.05 or *p* < 0.01.

## 5. Conclusions

A novel potential lectin gene (*ppa*) was cloned from *Pinellia pedatisecta*, and it shared 92.69% nucleotide similarity and 94% amino acid similarity with *Pinellia ternata* agglutinin (*pta*). The *ppa* gene could express in transgenic lines, and conferred them aphid-resistance indoors and in field conditions. Therefore, *ppa* could be a strong biotechnological candidate to produce aphid-resistant wheat.

## Abbreviations

PPA	*Pinellia pedatisecta* agglutinin
PTA	*Pinellia ternata* agglutinin
GNA	*Galanthus nivalis* agglutinin
ORF	Open Reading Frame;
DAF	Days After Flowering;
PCR	Polymerase Chain Reaction
RT-PCR	Reverse Transcriptional Polymerase Chain Reaction
